# *Rhodococcus aetherivorans* BCP1 as cell factory for the production of intracellular tellurium nanorods under aerobic conditions

**DOI:** 10.1186/s12934-016-0602-8

**Published:** 2016-12-15

**Authors:** Alessandro Presentato, Elena Piacenza, Max Anikovskiy, Martina Cappelletti, Davide Zannoni, Raymond J. Turner

**Affiliations:** 1 Microbial Biochemistry Laboratory, Department of Biological Sciences, University of Calgary, 2500 University Dr. NW, Calgary, AB T2N 1N4 Canada; 2 Department of Chemistry, University of Calgary, 2500 University Dr. NW, Calgary, AB T2N 1N4 Canada; 3 Department of Pharmacy and Biotechnology, Unit of General and Applied Microbiology, Via Irnerio 42, Bologna, 40126 Italy

**Keywords:** Tellurite, *Rhodococcus aetherivorans*, Elemental tellurium, Tellurium nanorods, Biogenic nanostructures, Nanorods biosynthesis

## Abstract

**Background:**

Tellurite (TeO_3_^2−^) is recognized as a toxic oxyanion to living organisms. However, mainly anaerobic or facultative-anaerobic microorganisms are able to tolerate and convert TeO_3_^2−^ into the less toxic and available form of elemental Tellurium (Te^0^), producing Te-deposits or Te-nanostructures. The use of TeO_3_^2−^-reducing bacteria can lead to the decontamination of polluted environments and the development of “green-synthesis” methods for the production of nanomaterials. In this study, the tolerance and the consumption of TeO_3_^2−^ have been investigated, along with the production and characterization of Te-nanorods by *Rhodococcus aetherivorans* BCP1 grown under aerobic conditions.

**Results:**

Aerobically grown BCP1 cells showed high tolerance towards TeO_3_^2−^ with a minimal inhibitory concentration (MIC) of 2800 μg/mL (11.2 mM). TeO_3_^2−^ consumption has been evaluated exposing the BCP1 strain to either 100 or 500 μg/mL of K_2_TeO_3_ (*unconditioned* growth) or after re-inoculation in fresh medium with new addition of K_2_TeO_3_ (*conditioned* growth). A complete consumption of TeO_3_^2−^ at 100 μg/mL was observed under both growth conditions, although *conditioned* cells showed higher consumption rate. *Unconditioned* and *conditioned* BCP1 cells partially consumed TeO_3_^2−^ at 500 μg/mL. However, a greater TeO_3_^2−^ consumption was observed with *conditioned* cells. The production of intracellular, not aggregated and rod-shaped Te-nanostructures (TeNRs) was observed as a consequence of TeO_3_^2−^ reduction. Extracted TeNRs appear to be embedded in an organic surrounding material, as suggested by the chemical–physical characterization. Moreover, we observed longer TeNRs depending on either the concentration of precursor (100 or 500 μg/mL of K_2_TeO_3_) or the growth conditions (*unconditioned* or *conditioned* grown cells).

**Conclusions:**

*Rhodococcus aetherivorans* BCP1 is able to tolerate high concentrations of TeO_3_^2−^ during its growth under aerobic conditions. Moreover, compared to *unconditioned* BCP1 cells, TeO_3_^2−^ *conditioned* cells showed a higher oxyanion consumption rate (for 100 μg/mL of K_2_TeO_3_) or to consume greater amount of TeO_3_^2−^ (for 500 μg/mL of K_2_TeO_3_). TeO_3_^2−^ consumption by BCP1 cells led to the production of intracellular and not aggregated TeNRs embedded in an organic surrounding material. The high resistance of BCP1 to TeO_3_^2−^ along with its ability to produce Te-nanostructures supports the application of this microorganism as a possible eco-friendly nanofactory.

**Electronic supplementary material:**

The online version of this article (doi:10.1186/s12934-016-0602-8) contains supplementary material, which is available to authorized users.

## Background

Tellurium (Te) was discovered by Franz-Joseph Müller von Reicheinstein in 1782 [1], and in nature this element can be found in gold ores as association with metals, forming calaverite (AuTe_2_), sylvanite (AgAuTe_4_) and nagyagite [AuPb(Sb, Bi)Te_2–3_S_6_] [2]. Te is an element of the chalcogen family, belonging to the Group 16 of the periodic table along with oxygen (O), sulfur (S), selenium (Se), and the radioactive element polonium (Po) [3]. Additionally, it is defined as a metalloid due to its intermediate properties between metals and non-metals [3]. Due to the anthropogenic activity, Te is normally present in the environment as inorganic telluride (Te_2_), the oxyanions tellurite (TeO_3_^2−^) and tellurate (TeO_4_^2−^), and the organic dimethyl telluride (CH_3_TeCH_3_) [4]. Among these, TeO_3_^2−^ is the most soluble form of tellurium, and it is the most toxic form for both prokaryotes and eukaryotes [5] at concentrations as low as 1 μg/mL [6]. This concentration is several orders of magnitude lower as compared to others metals and metalloids of public health and environmental concern such as selenium, iron, mercury, cadmium, copper, chromium, zinc, and cobalt [7, 8]. Furthermore, due to tellurite’s use in electronics as well as industrial glasses, it can be found highly concentrated in soil and water near waste discharge sites of manufacturing and processing facilities [9], as a hazardous and toxic pollutant [6]. Despite TeO_3_^2−^ toxicity, several Gram-negative microorganisms capable to grow phototrophycally or chemotrophycally under aerobic and anaerobic conditions have been described for their capability to reduce this toxic oxyanion, such as *Rhodobacter capsulatus* B100, *Shewanella odeinensis* MR-1, *Pseudomonas pseudoalcaligenes* KF707, and *Escherichia coli* HB101 strain [10–13]. Additionally, α-Proteobacteria resistant to concentrations of TeO_3_^2−^ ranging from 1 to 25 mg/mL [14, 15] and a few Gram-positive strains (e.g., *Bacillus beveridgei* sp.nov., *Bacillus selenitireducens*, *Corynebacterium diphtheria*, *Lysinibacillus* sp. ZYM-1, *Bacillus* sp. BZ, *Bacillus* sp. STG-83, *Paenibacillus* TeW, and *Salinicoccus* sp. QW6) resistant to low level of TeO_3_^2−^ (ranging from 0.2 to 3 mg/mL) were also reported [16–23].

It has been established that TeO_3_^2−^-reducing bacteria are able to convert this oxyanion to the less toxic elemental tellurium (Te^0^), which is cytosolically accumulated as black inclusions [6] and/or defined nanostructures such as nanocrystals, nanorods (NRs) and nanoparticles (NPs) [24]. Particularly, Kim and colleagues [25] showed the capability of *Shewanella oneidensis* MR-1 to produce tellurium nanorods (TeNRs), while *Rhodobacter capsulatus* B100 is able to produce both intra- and extra-cellular needle-shaped Te-nanocrystals [10]. Another example is the synthesis of tellurium nanoparticles (TeNPs) in cells of *Ochrobactrum* MPV-1 [26].

NPs and NRs have different physical–chemical and biological properties compared to their bulk counterparts, due to their size, high surface–volume ratio, large surface energy and spatial confinement, allowing the use of these nanostructures in biomedical, electronic, environmental, and renewable energy fields, to name a few [24]. In this context, the natural ability of microorganisms to generate nanostructures by the reduction of toxic oxyanions can play two key roles: (1) the development of eco-friendly “green-synthesis” methods for the production of NPs or NRs [27], and (2) the decontamination of metal polluted environments [28]. Moreover, the biological synthesis of either NPs or NRs has several advantages over the chemical one, namely: (1) it does not require the use of toxic chemicals; (2) it does not result in the formation of hazardous wastes; and (3) it has a substantial lower cost of production [29].

Strains of the *Rhodococcus* genus, belonging to the Mycolata group of *Actinomycetes*, are aerobic non-sporulating bacteria, which are ideal microorganisms for bioremediation and industrial uses due to their remarkable capacity to catalyze a very wide range of compounds and their environmental robustness [30]. Although the ability of *Rhodococcus* spp. to degrade xenobiotics along with their physiological adaptation strategies, i.e. cell membrane composition and intracellular inclusions, were largely reported in the literature [31], much less is known about the *Rhodococcus* genus capacity to resist to toxic metals/metalloids. In this respect, *Rhodococcus aetherivorans* BCP1, a hydrocarbon- and chlorinated solvent degrader that was recently described for its unique capacity to overcome stress environmental conditions in the presence of a wide range of antimicrobials and toxic metals/metalloids such as tellurite, arsenate and selenite [32–36] appears to be an interesting candidate to study. Thus, the present work investigates the ability of *Rhodococcus aetherivorans* BCP1 to survive in the presence of increasing concentrations of tellurite and to produce Te-nanostructures. In particular, we evaluated the capacity of BCP1 strain to grow in the presence of high concentrations of TeO_3_^2−^ oxyanions supplied as K_2_TeO_3_. TeO_3_^2−^ consumption rates were also assessed after re-inoculation of pre-exposed cells in fresh medium with new addition of K_2_TeO_3_ (*conditioned* cells). Finally, the production of Te-nanostructures was investigated through the use of physical–chemical methods.

## Methods

### Bacterial strain, growth media, culture conditions

The strain *Rhodococcus aetherivorans* BCP1 (DSM 44980) was pre-cultured in 250 mL Erlenmeyer Baffled Flask for 2 days, containing 25 mL of Luria–Bertani medium (here indicated as LB) [composed of (g/L) NaCl, 10; Yeast Extract, 5; Tryptone, 10]. When necessary, the medium was solidified by adding 15 g/L of Agar. BCP1 cells were then inoculated (1% v/v) and grown for 5 days in 50 mL of LB medium supplied with either 100 (0.4 mM) or 500 (2 mM) µg/mL of K_2_TeO_3_. Here we refer to this first bacterial growth as *unconditioned*. After this growth step, BCP1 cells were re-inoculated (1% v/v) and cultured for other 5 days in 50 mL of fresh LB medium and 100 or 500 µg/mL of K_2_TeO_3_. This secondary bacterial growth is here defined as *conditioned*. Each culture was incubated aerobically at 30 °C with shaking (150 rpm). In order to evaluate the bacterial growth rate, every 24 h an aliquot (100 µL) of BCP1 cells was collected from each culture and serially diluted in sterile saline solution (NaCl 0.9% w/v). The cells were recovered on LB agar plates for 48 h at 30 °C. The number of growing cells is reported as average of the Colony Forming Unit per milliliter (CFU/mL) counted for each biological trial (n = 3) with standard deviation. All the reagents were purchased from Sigma-Aldrich^®^.

### Evaluation of TeO_3_^2−^ minimal inhibitory concentration (MIC)

In order to establish the minimal inhibitory concentration (MIC) of tellurite, i.e. as the concentration of K_2_TeO_3_ at which no bacterial growth was observed, the BCP1 strain was exposed to concentrations of K_2_TeO_3_ ranging from 100 to 3000 µg/mL (0.4–12 mM). After 24 h of incubation the number of viable cells was determined by spot plates count on LB agar recovery plates. The assay was conducted in triplicate and the data are reported as average of the CFU/mL counted with standard deviation. The established MIC and corresponding kill curve was used to choose the best concentration of K_2_TeO_3_ to use for nano-material production.

### TeO_3_^2−^ consumption assay

The residual concentration of TeO_3_^2−^ oxyanions in the culture broth was estimated as described elsewhere [37]. Briefly, 1 mL of BCP1 cells grown as *unconditioned* or *conditioned* in the presence of K_2_TeO_3_ was collected every 12 up to 120 h. The sample was centrifuged at 14,000 rpm for 2 min in order to separate the bacterial cell pellet from the supernatant, and a 10–100 µL aliquot was mixed with 600 µL of 0.5 M Tris–HCl buffer pH 7.0 (VWR^®^), 200 µL of diethyldithiocarbamate (Sigma-Aldrich^®^), and LB up to a total volume of 1 mL. The absorbance of the mixture was read at 340 nm using a Varian Cary^®^ 50 Bio UV–Visible Spectrophotometer. The residual concentration of TeO_3_^2−^ oxyanions was determined using this absorbance values and the calibration curve obtained for known concentrations (0, 10, 20, 30, 40, 50 and 60 µg/mL) of K_2_TeO_3_ in LB (R^2^ = 0.99). The data are reported as average values (n = 3) with standard deviation.

### Preparation, extraction, and purification of TeNRs

In order to extract and purify TeNRs produced by the BCP1 strain grown as *unconditioned* or *conditioned* cells, biomasses were collected by centrifugation (3700 rpm) for 20 min after 5 culturing days. The pellets were washed twice with saline solution (NaCl 0.9% w/v) and resuspended in Tris–HCl (1.5 mM) buffer pH 7.4. Bacterial cells were disrupted by ultrasonication at 22 W for 10 min (30 s burst interspersed by 30 s of pause) on ice (MICROSON™ Ultrasonic Cell Disruptor XL, Qsonica Misonix Inc.). The cellular debris was then separated from TeNRs in the supernatant by a centrifugation step (3700 rpm) for 20 min. Supernatants containing TeNRs were incubated overnight (16 h) at 4 °C with 1-Octanol (Sigma-Aldrich^®^) in a ratio 4:1 (v/v) and then recovered by centrifugation (16,000 rpm) for 15 min. TeNRs pellets were finally suspended in deionized water.

Here we refer to the TeNRs produced by the BCP1 strain as TeNRs_100_ or TeNRs_500_, depending on the initial concentration of K_2_TeO_3_ present in the growth medium.

### Dynamic light scattering (DLS) and zeta potential measurements

DLS and zeta potential measurements of TeNRs produced by BCP1 cells grown as *unconditioned* or *conditioned* were performed using a Zen 3600 Zetasizer Nano ZS™ from Malvern Instruments. The samples (1 mL each) were analyzed in a spectrophotometric cuvette (10 × 10 × 45 mm Acrylic Cuvettes, Sarstedt) and in a folded capillary Zeta cell (Malvern Instruments) for DLS and zeta potential measurements, respectively.

### Transmission electron microscopy (TEM) analysis

TEM observations of TeNRs extracted from BCP1 cells grown as *unconditioned* or *conditioned* were carried out by mounting 5 µL of each sample on carbon-coated copper grids (CF300-CU, Electron Microscopy Sciences), air-drying the samples, and imaging them using a Hitachi H7650 TEM. The distribution of TeNRs length was calculated by measuring the length of 100 randomly chosen nanorods through the use of ImageJ software. The distribution was fitted to a Gaussian function to yield the average length. In order to image BCP1 cells grown in the presence of 100 or 500 µg/mL K_2_TeO_3_ for 5 days, the cells were negatively stained using a 1% phosphotungstic acid solution (pH 7.3).

### Scanning electron microscopy (SEM) and energy-dispersed X-ray spectroscopy (EDX) analysis

The samples were prepared by depositing TeNRs suspensions onto Crystal Silicon wafers (type N/Phos, size 100 mm, University Wafer) and air-drying. Imaging and EDX analysis were performed on a Zeiss Sigma VP scanning electron microscope and an Oxford Instruments INCAx-act system, respectively.

## Results

### Minimal inhibitory concentration (MIC) assay of *Rhodococcus* sp. BCP1 strain

**Fig. 1:**
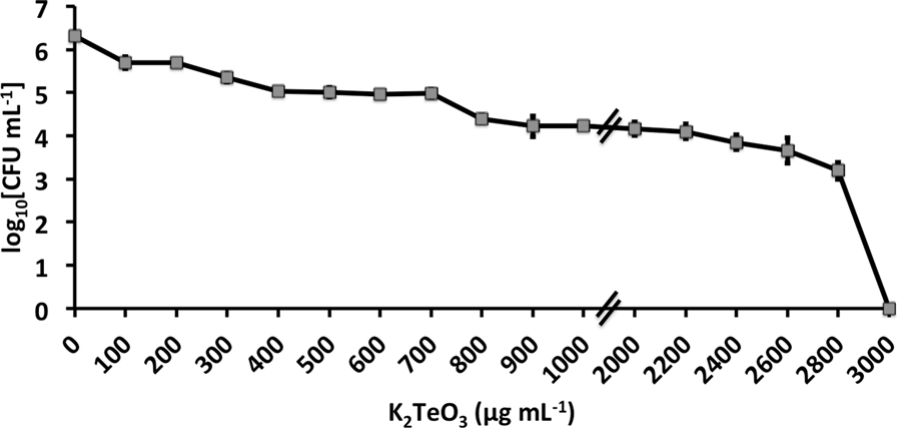
Kill curve of *Rhodococcus* *aetherivorans* BCP1 exposed for 24 h to increasing concentration of K_2_TeO_3_, with the established minimal inhibitory concentration (MIC)

In order to evaluate the BCP1 strain’s ability to tolerate TeO_3_^2−^ oxyanions present in the growth medium (LB), the MIC was established by exposing the cells for 24 h to different K_2_TeO_3_ concentrations, ranging from 0 to 3000 µg/mL (0–12 mM). The data are plotted in Fig. 1 as a kill curve displaying the number of BCP1 viable cells against the K_2_TeO_3_ concentration values. As a result, the MIC value of TeO_3_^2−^ was estimated at 2800 µg/mL (11.2 mM) that corresponded to 3 log reduction as compared to the number of viable cells counted at the time of inoculation, while only 1 and 2 log reduction of BCP1 viable cells was observed when the K_2_TeO_3_ was varied from 100 to 1000 µg/mL (0.4–4 mM) and from 100 to 2000 µg/mL (0.4–8 mM), respectively.

### Growth and consumption of TeO_3_^2−^ by the BCP1 strain, and localization of TeNRs

**Fig. 2:**
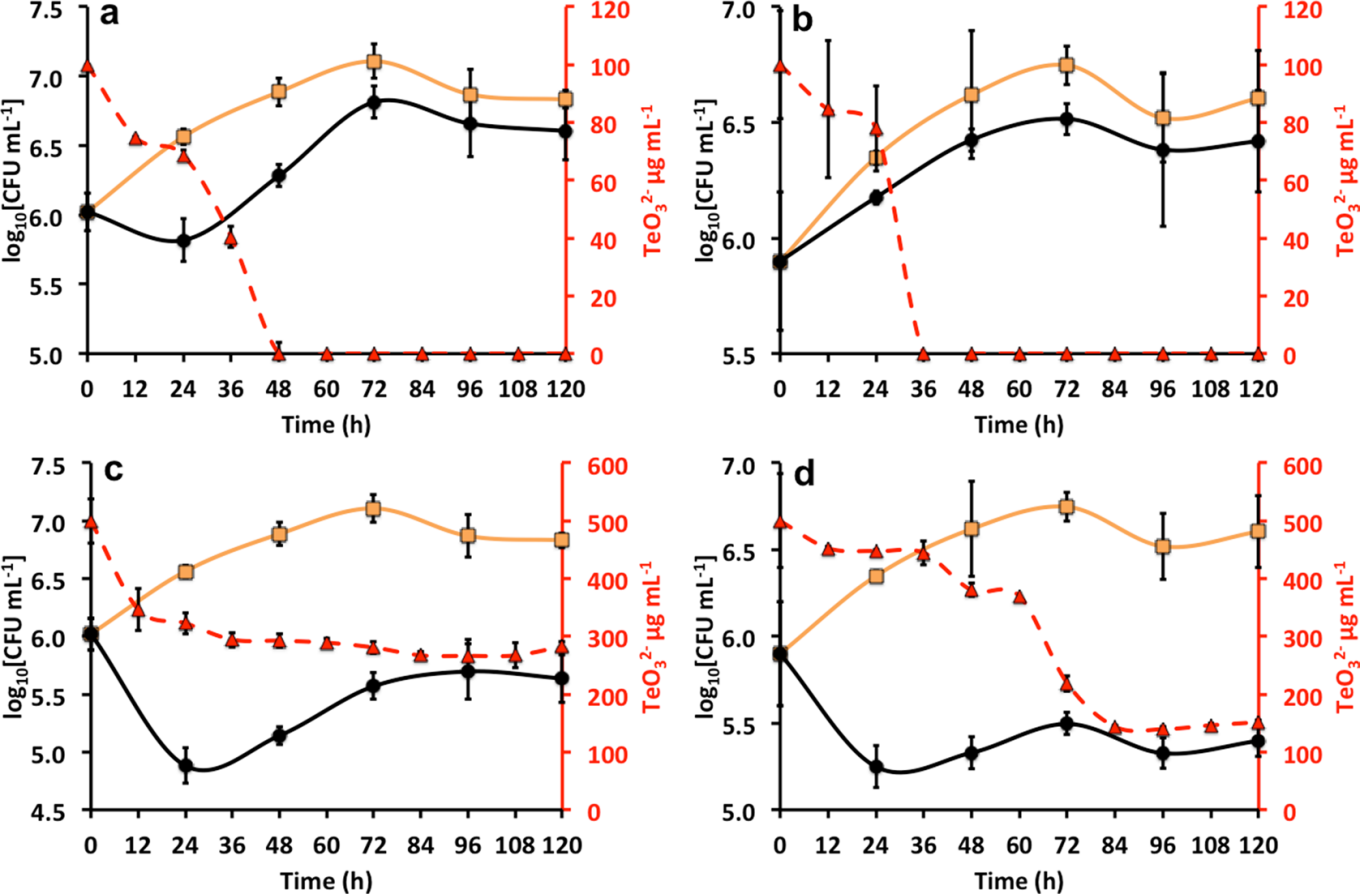
*Rhodococcus* *aetherivorans* BCP1 growth in  LB medium,  LB supplied with 100 or 500 µg/mL of K_2_TeO_3_ as *unconditioned* (**a**, **c**) or *conditioned* (**b**, **d**) cells, and  TeO_3_^2−^ consumption

Since the number of BCP1 viable cells decreased by less than 1 log after 24 h exposure to 100 µg/mL (5.00 × 10^5^ CFU/mL) or 500 µg/mL (1.00 × 10^5^ CFU/mL) of K_2_TeO_3_, the growth and consumption of TeO_3_^2−^ at these concentrations by the BCP1 strain were evaluated for both *unconditioned* and *conditioned* grown cells (Fig. 2). *Unconditioned* BCP1 cells grown in the presence of 100 µg/mL of K_2_TeO_3_ showed an initial consumption of the oxyanions during their lag phase (24 h), while a complete reduction occurred in the early exponential growth phase (48 h), showing a stationary phase after 60 h of growth (Fig. 2a). In the case of *conditioned* BCP1 cells the reduction of the same amount of TeO_3_^2−^ was 12 h faster (36 h) as compared to those grown as *unconditioned*, occurring in the early exponential growth phase. As for *unconditioned* cells, the *conditioned* ones reached the stationary phase after 60 h of incubation and any lag phase of growth was observed (Fig. 2b). By contrast, considering *unconditioned* BCP1 cells growing in the presence of 500 µg/mL of K_2_TeO_3_, the consumption/reduction of the oxyanions was not complete over the incubation time (120 h), resulting in the reduction of about 45% (218 µg) of the initial amount of TeO_3_^2−^ (Fig. 2c). Particularly, the initial amount of the oxyanions decreased by 153 µg during the lag phase of growth (24 h), reaching the maximum extent of reduction after 72 h of incubation (282 µg), and it remained constant over the stationary growth phases (Fig. 2c). Regarding *conditioned* BCP1 K_2_TeO_3_-grown cells in the presence of 500 µg/mL, we did not observe a complete reduction of the initial TeO_3_^2−^ concentration, although the amount of residual oxyanions present in the medium was lower (152 µg) as compared to *unconditioned* grown cells. Specifically, a reduction of 56 µg of TeO_3_^2−^ oxyanions during the initial 36 h of incubation was observed, which corresponds to the lag phase of growth, while after 84 h TeO_3_^2−^ oxyanions concentration dropped down to its minimal value, along with an actual growth of the biomass (Fig. 2d).

**Fig. 3:**
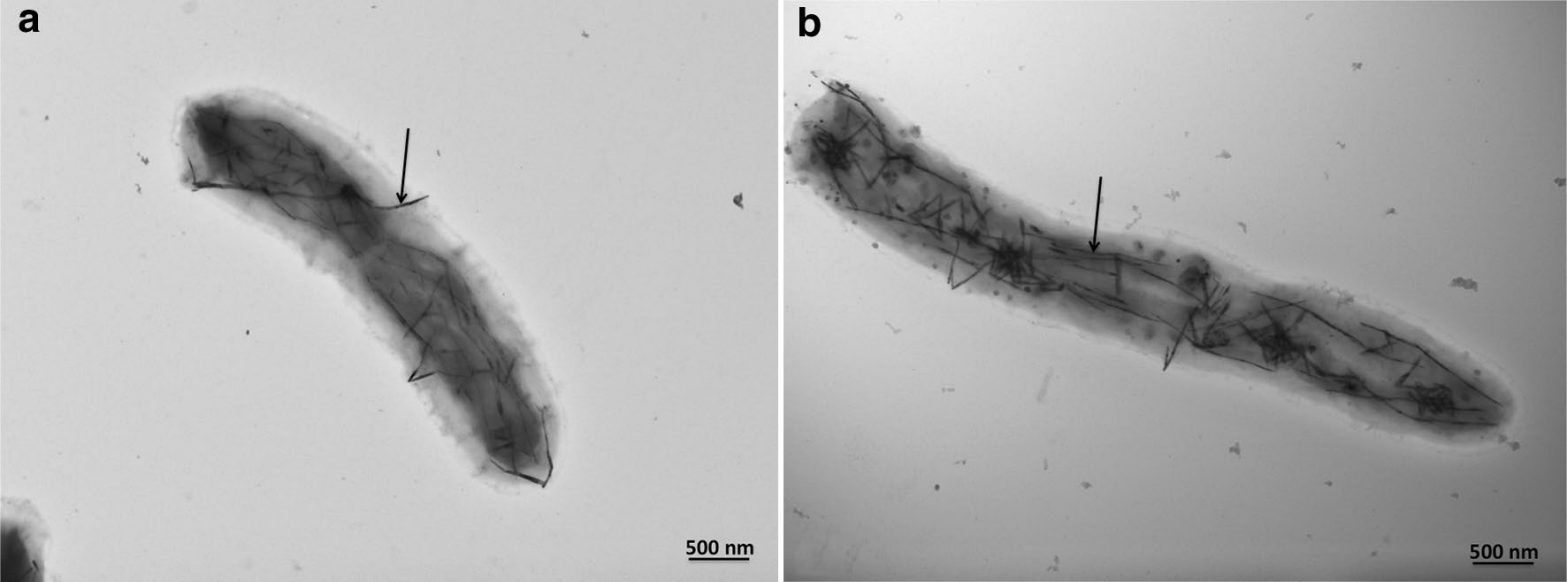
Transmission electron microscopy (TEM) micrographs of BCP1 cells grown for 120 h in the presence of 100 µg/mL (**a**), and 500 µg/mL (**b**) of K_2_TeO_3_. *Arrows* indicate the intracellular TeNRs produced by the BCP1 strain

To detect the production of tellurium nanostructures by BCP1, either 100 or 500 µg/mL K_2_TeO_3_-grown cells for 5 days were negatively stained and analyzed by TEM (Fig. 3). In both cases, the presence of intracellular TeNRs was detected (Fig. 3a, b).

### Dynamic light scattering (DLS) analyses

DLS experiments were performed on TeNRs extracted from BCP1 *unconditioned* and *conditioned* grown cells (Additional file 1: Figure S1). The measurements yielded distributions of sizes centered at 295 nm (Additional file 1: Figure S1a, b) for the samples of TeNRs_100_ produced by BCP1 strain grown as *unconditioned* or *conditioned* cells, with a standard deviation of ±61 nm (*unconditioned*) and ±22 nm (*conditioned*). TeNRs_500_ isolated from *unconditioned* and *conditioned* grown cells were featured by a size distribution centered at 342 nm (Additional file 1: Figure S1c, d), with a standard deviation of ±64 and ±86 nm, respectively. The TeNRs populations were found to be polydisperse as indicated by the values of the measured polydispersity index, being 0.398 (TeNRs_100_) and 0.395 (TeNRs_500_) for Te-nanostructures generated by *unconditioned* BCP1 cells, and 0.384 (TeNRs_100_) and 0.381 (TeNRs_500_) for those isolated from *conditioned* cells. Additional DLS experiments were performed on the supernatants containing TeNRs, which were recovered by removing TeNRs from the samples through centrifugation at 8000 rpm for 10 min. The DLS measurements performed on the supernatants (Additional file 1: Figure S2) produced distributions shifted towards smaller sizes compared to the ones obtained from the samples containing the nanorods (Additional file 1: Figure S1): 142 ± 14 and 164 ± 9 nm (Additional file 1: Figure S2a, b) for the supernatants recovered after removing TeNRs_100_ produced by BCP1 grown as *unconditioned* or *conditioned* cells, and 142 ± 17 and 122 ± 12 nm (Additional file 1: Figure S2c, d) for the supernatants obtained after removing TeNRs_500_ generated by the cells grown as *unconditioned* or *conditioned*, respectively. As a control, DLS analysis of the supernatant derived from the BCP1 culture grown for 120 h on rich medium (LB) showed a peak centered at 1 ± 0.48 nm (Additional file 1: Figure S2e), which is likely due to the presence of peptides in the culture broth.

### Transmission electron microscopy (TEM) analysis and size distribution of TeNRs

**Fig. 4:**
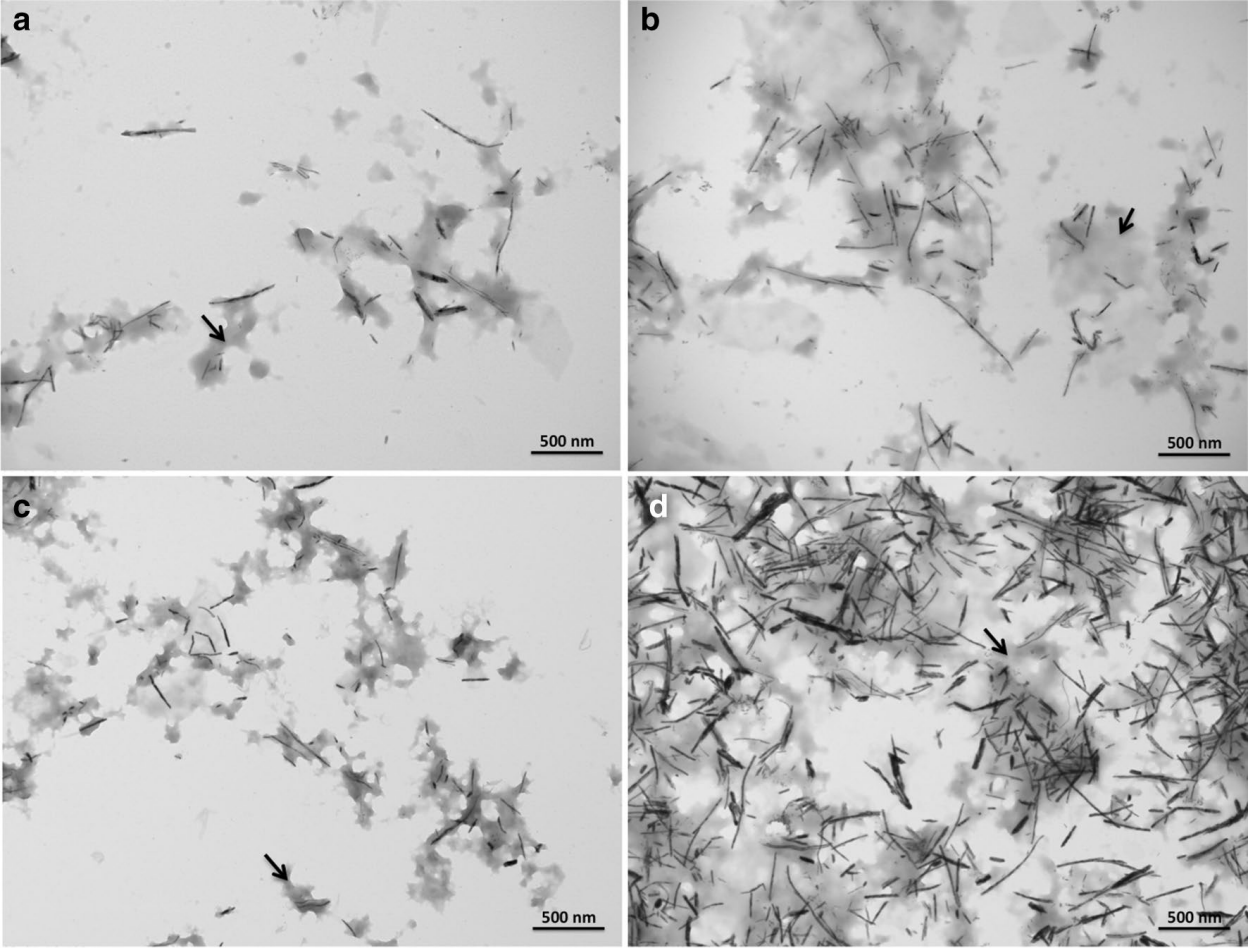
Transmission electron microscopy (TEM) micrographs of TeNRs_100_ (**a**), and TeNRs_500_ (**b**) extracted from the BCP1 strain grown as *unconditioned* cells in the presence of K_2_TeO_3_, and TeNRs_100_ (**c**), and TeNRs_500_ (**d**) recovered from those *conditioned*

**Fig. 5:**
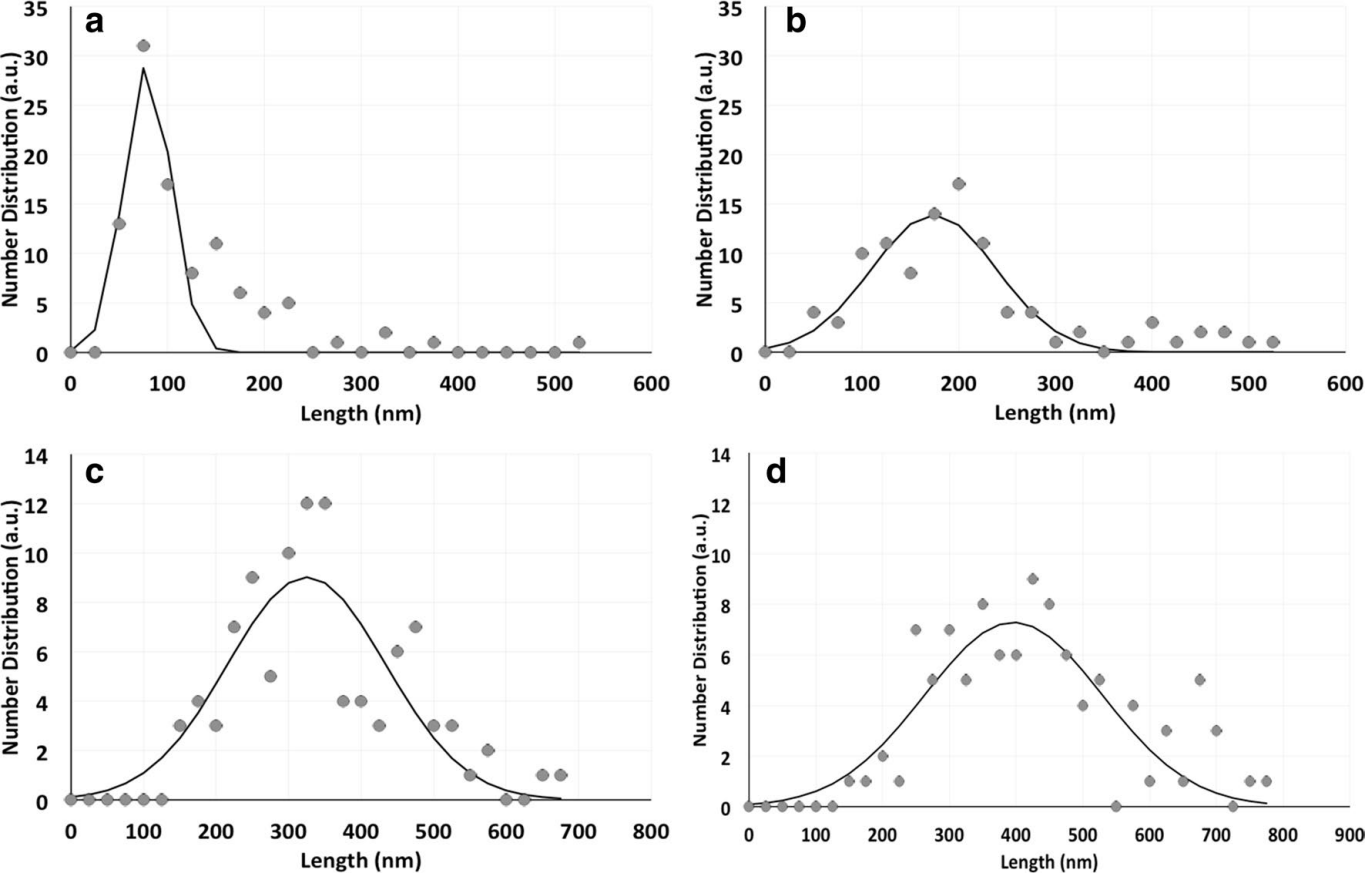
Length distribution (nm) of TeNRs_100_ (**a**), and TeNRs_500_ (**b**) generated by *unconditioned* BCP1 K_2_TeO_3_-grown cells, and TeNRs_100_ (**c**), and TeNRs_500_ (**d**) isolated from *conditioned* ones. Length distributions are indicated as *grey filled circles*, while the Gaussian fit is highlighted as a continuous *black curve*

TEM observations were carried out on extracted TeNRs in order to study the size and morphology of TeNRs produced by both *unconditioned* and *conditioned* cells (Fig. 4). TeNRs from *unconditioned* cells revealed the presence of electron-dense and not aggregated NRs showing variability in length (Fig. 4a, b). Particularly, the length measurements using ImageJ software of 100 randomly chosen NRs yielded an average size of 148 ± 104 and 223 ± 116 nm for TeNRs_100_ and TeNRs_500_, respectively (Fig. 5a, b). High electron-density was observed in TeNRs extracted from *conditioned* cells as well (Fig. 4c, d). TeNRs_100_ or TeNRs_500_ isolated from BCP1 *conditioned* cells were longer compared to those generated by *unconditioned* cells, with a broader length distribution. In this case, the evaluated average size of NRs is 354 ± 125 and 463 ± 147 nm for TeNRs_100_ and TeNRs_500_, respectively (Fig. 5c, d). Furthermore, the TEM analyses of TeNRs extracted from either *unconditioned* or *conditioned* cells revealed the presence of an electron-dense material surrounding the nanorods (Fig. 4, indicated by arrows).

### Zeta potential measurement

Zeta potential measurements were conducted to evaluate whether the surface of TeNRs was charged (Additional file 1: Figure S3). A single peak at −25 mV was detected in Zeta potential plots for both *unconditioned* generated TeNRs_100_ and TeNRs_500_ (Additional file 1: Figure S3a, b). The zeta potential results obtained for TeNRs produced by *conditioned* BCP1 cells indicated the presence of a less negative potential (−20 mV) in the case of TeNRs_100_, while TeNRs_500_ were featured by the same potential value of *unconditioned* NRs (−25 mV) (Additional file 1: Figure S3c, d). Similarly to the DLS analysis, additional zeta potential measurements were performed on the supernatants recovered after removing TeNRs through centrifugation (Additional file 1: Figure S4), resulting in similar surface potential values as compared to those obtained for TeNRs suspensions. Particularly, the supernatants recovered from TeNRs produced by *unconditioned* cells grown in the presence of either 100 or 500 µg/mL of K_2_TeO_3_ were featured by a surface potential of −26 and −22 mV (Additional file 1: Figure S4a, b), while those obtained from TeNRs_100_ and TeNRs_500_ generated by *conditioned* cells had a charge of −29 and −21 mV (Additional file 1: Figure S4c, d), respectively.

### Scanning electron microscopy (SEM) and energy-dispersed X-ray spectroscopy (EDX) analyses

**Fig. 6:**
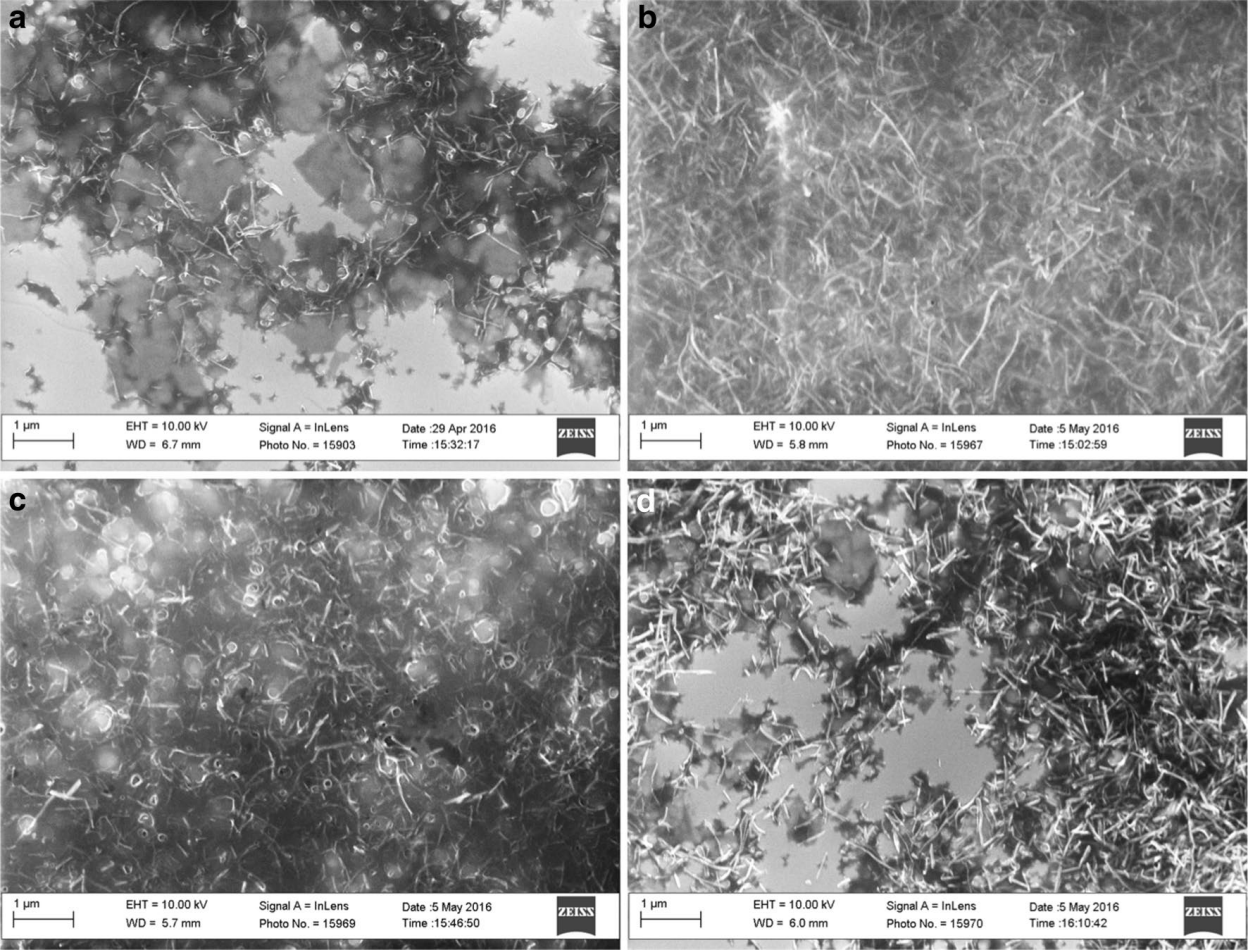
Scanning electron microscopy (SEM) micrographs of TeNRs_100_ (**a**), and TeNRs_500_ (**b**) produced by *unconditioned* BCP1 K_2_TeO_3_-grown cells, and TeNRs_100_ (**c**), and TeNRs_500_ (**d**) extracted from those *conditioned*

**Fig. 7:**
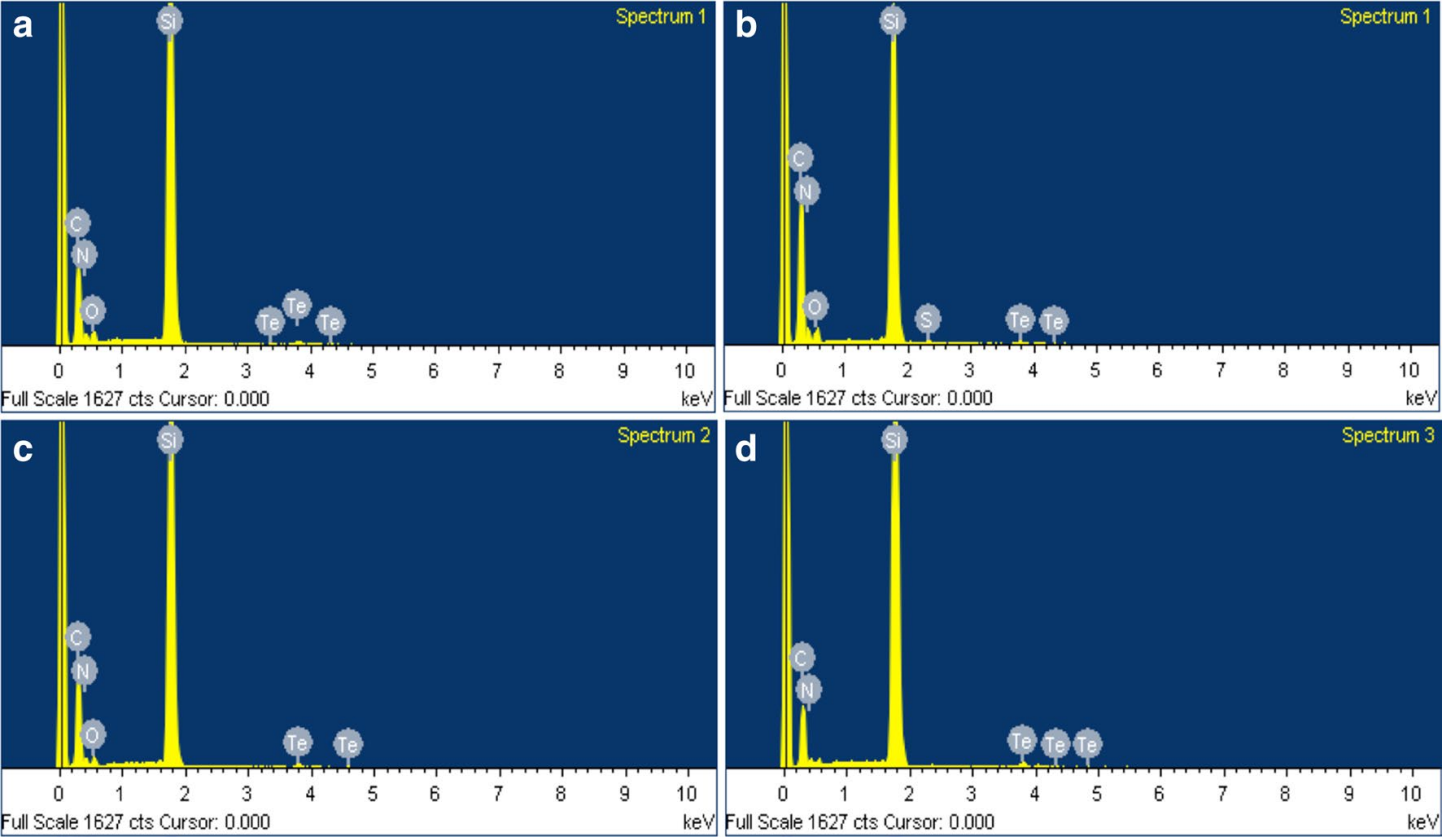
Energy-dispersed X-ray spectroscopy (EDX) spectra of TeNRs_100_ (**a**), and TeNRs_500_ (**b**) *unconditioned* BCP1 grown cells, and TeNRs_100_ (**c**), and TeNRs_500_ (**d**) extracted from those *conditioned* ones grown in the presence of K_2_TeO_3_

Morphology of TeNRs extracted from BCP1 *unconditioned* and *conditioned* cells was evaluated by performing SEM observations (Fig. 6), while the elemental analysis of NRs was performed using energy-dispersed X-ray spectroscopy (EDX) (Fig. 7; Table 1). SEM images showed the presence of not aggregated TeNRs surrounded by a dark grey colored material in background (Fig. 6) similarly to TEM observations. In particular, TeNRs_100_ recovered from *unconditioned* cells underlined the evidence of some NRs forming circular structures around the edge of the surrounding material, while the TeNRs_500_ were homogeneously distributed and had a rod-shaped morphology (Fig. 6a, b). Elemental analysis of TeNRs showed the presence of the same chemical elements for different initial concentrations of the precursor (K_2_TeO_3_): carbon, nitrogen, oxygen and tellurium (Fig. 7a, b). However, the relative percentage ratios of these elements differed between the TeNRs_100_ and TeNRs_500_. The presence of silicon in the elemental analysis was due to the silicon stubs the samples were mounted onto. Excluding the silicon signal, carbon had the highest percentage value in both TeNRs extracted from *unconditioned* cells, being 39% (TeNRs_100_) and 49.7% (TeNRs_500_). EDX quantification data showed a higher amount of nitrogen for TeNRs_500_ (9%) as compared to TeNRs_100_ (5%), while oxygen percentage values were comparable for *unconditioned* TeNRs, yielding 4% (TeNRs_500_) and 3% (TeNRs_100_). Similarly, tellurium amounts were comparable between TeNRs_100_ (4%) and TeNRs_500_ (3%). Moreover, low content of sulfur (0.3%) was detected only in the case of TeNRs_500_ (Table 1). SEM observations of TeNRs produced by *conditioned* cells revealed morphologies analogous to those seen in *unconditioned* cells, with the presence of circular organized NRs in the case of TeNRs_100_ and the typical rod-morphology for TeNRs_500_ (Fig. 6c, d). Chemical composition detected by EDX analyses of these nanostructures recovered from *conditioned* cells indicated the presence of carbon, nitrogen and tellurium (Fig. 7c, d). Carbon showed the highest relative percentage value, being 42% (TeNRs_100_) and 34% (TeNRs_500_), while nitrogen amounts were higher in TeNRs_100_ (7%) than TeNRs_500_ (3%). Moreover, tellurium percentages underlined a relative value of 6 and 3% in TeNRs_500_ and TeNRs_100_, respectively. Finally, only in the case of TeNRs_500_, EDX data showed the absence of the oxygen signal, which was detected in low content (3%) in TeNRs_100_ (Table 1).

**Table 1:** Elemental quantification (as weight relative percentage) of *unconditioned* and *conditioned* TeNRs_100_ and TeNRs_500_

Element	*Unconditioned*		*Conditioned*	
	TeNRs_100_	TeNRs_500_	TeNRs_100_	TeNRs_500_
	Weight (Rel. %)	Weight (Rel. %)	Weight (Rel. %)	Weight (Rel. %)
Silicon (Si)	49	34	45	57
Tellurium (Te)	4	3	3	6
Carbon (C)	39	49.7	42	34
Oxygen (O)	3	4	3	N.D.
Nitrogen (N)	5	9	7	3
Sulfur (S)	N.D.	0.3	N.D.	N.D.

Elemental quantification is expressed as Weight Relative Percentage of the element detected in the TeNRs samples

Element not detected are indicated as N.D

## Discussion

Although Te is a rare natural element in the Earth crust (0.027 ppm) [12], the widespread use of Te-containing compounds in electronics, optics, production of batteries, petroleum refining and mining [12, 38–40] has led to an increase in its presence in the environment as soluble and toxic oxyanion TeO_3_^2−^, causing serious threats to the ecosystem and human health [28]. Interestingly, a large number of Gram-negative [10–13] and Gram-positive bacteria [16–18] were reported to be tolerant and/or resistant towards tellurite. A common strategy used by microorganisms to overcome the toxicity of TeO_3_^2−^, relies on the reduction of this oxyanion to its less available/toxic elemental form (Te^0^), producing either intracellular metalloid deposits or nanostructures [12]. In this present study, we have evaluated the capacity of an aerobic Gram-positive *Rhodococcus* strain, *Rh. aetherivorans* BCP1, to grow in the presence of high amounts of tellurite (supplied as K_2_TeO_3_). The results show that under this extreme growth condition, BCP1 cells are able not only to grow significantly but they also reduce TeO_3_^2−^ generating intracellular Te-nanostructures, which were isolated and characterized. This result is of some importance since in the past it was reported that oxygen greatly enhances the TeO_3_^2−^ toxicity to bacterial cells, i.e. from MIC^Te^ of 250 to 2 µg/mL under anaerobic and aerobic growth, respectively [41]. Conversely, the tolerance of aerobically grown BCP1 strain towards TeO_3_^2−^ oxyanions was very high, with a MIC^Te^ value of 2800 µg/mL (11.2 mM). A comparison between BCP1 strain and Gram-positive bacteria described in literature for their ability to grow aerobically in the presence of K_2_TeO_3_ underlines the high tolerance of *Rhodococcus aetherivorans* BCP1 strain to this oxyanion. Specifically, bacterial strains such as *Lysinibacillus* sp. ZYM-1, *Bacillus* sp. BZ, *Corynebacterium diphtheriae*, *Bacillus* sp. STG-83, *Paenibacillus* TeW, and *Salinicoccus* sp. QW6 were described for their ability to tolerate TeO_3_^2−^, with an MIC^Te^ values ranging from 0.8 to 12 mM [18–23] (Table 2).

**Table 2:** Comparison of the minimal inhibitory concentration of tellurite (MIC^Te^) supplied as potassium tellurite (K_2_TeO_3_) to rich medium among Gram-positive bacteria grown under aerobic conditions

Strain	MIC^Te^ (mM)	References
*Salinicoccus* sp. QW6	12	Amoozegar et al. [23]
*Rhodococcus aetherivorans* BCP1	11.2	This study
*Lysinibacillus* sp. ZYM-1	2	Zhao et al. [19]
*Bacillus* sp. STG-83	1.25	Soudi et al. [21]
*Corynebacterium diphtheriae*	1	Tucker et al. [18]
*Paenibacillus* TeW	1	Chien et al. [22]
*Bacillus* sp. BZ	0.8	Zare et al. [20]

Among the species of *Actinomycetales* order, BCP1 strain tolerance is therefore ten times higher than the MIC^Te^ (1 mM) of *Corynebacterium diphtheriae* [18]. Conversely, the MIC^Te^ of BCP1 strain was comparable to that obtained with *Salinicoccus* sp. QW6, which is equal to 12 mM [23]. In this respect, the high tolerance of the BCP1 cells towards TeO_3_^2−^ oxyanions under aerobic conditions suggests that this microorganism might play a key role in the in situ and/or ex situ decontamination procedures of TeO_3_^2−^ polluted environments.

In order to evaluate differences in the growth, in the reduction of TeO_3_^2−^, as well as in the production of TeNRs by BCP1 strain, *unconditioned* and *conditioned* cells were exposed to either 100 or 500 µg/mL (0.4 or 2 mM) K_2_TeO_3_. The complete reduction of 100 µg/mL TeO_3_^2−^ to elemental Te^0^ within 36 h was observed for *conditioned* BCP1 grown cells as compared to the *unconditioned* ones (48 h). Similarly, Amoozegar et al. [23] observed that *Salinicoccus* sp. QW6 was able to completely reduce 0.5 mM (125 µg/mL) of K_2_TeO_3_ within 72 h under aerobic conditions. There was no increased removal detected by the QW6 strain at greater concentrations, even after 144 h of incubation. Additionally, an incomplete reduction of TeO_3_^2−^ was described by Zare et al. [20] in the case of *Bacillus* sp. BZ incubated in Nutrient Broth medium supplemented with 50 or 100 µg/mL (0.2 or 0.4 mM) of K_2_TeO_3_ within 50 h of exposure. By contrast, when the BCP1 strain was incubated in the presence of 500 µg/mL of K_2_TeO_3_, the reduction of the initial concentration of TeO_3_^2−^ oxyanions resulted to be higher in the case of BCP1 *conditioned* grown cells (348 µg) rather than the *unconditioned* ones (218 µg), within 5 culturing days. Nevertheless, an incomplete reduction of the TeO_3_^2−^ added (500 µg/mL) was observed. Although cellular thiols (RSH) and glutathione (GSH) molecules are likely to reduce TeO_3_^2−^ oxyanions [5] with a consequence of a strong cytoplasmic redox unbalance of the glutathione/glutaredoxin and thioredoxin pool [42, 43], it is noteworthy that glutathione molecules are not commonly present in *Actinobacteria*, except in the case of horizontal gene transfer [44]. In *Actinomycetes* strains, analogous functions to glutathione (GSH) molecules are performed by mycothiols (MSH; also designated AcCys-GlcN-Ins), which are the major species of thiols present [45]. Similarly to GSHs, MSHs are able to reduce metals and toxic compounds thanks to the presence of thiol groups in cysteine moieties [45], which provide three possible metal ligands (–S^−^, –NH_2_, –COO^−^). The result of these oxidation–reduction reactions is the production of reactive oxygen species (ROS) e.g. hydrogen peroxide, which cause cellular death [46]. On the other hand, both GSH and MSH molecules are less prone to the oxidation when amino and carboxylic groups are blocked by γ-glutamyl and glycine residues or acetyl and GlcN-Ins, respectively [47, 48]. In this respect, the capacity of BCP1 cells to grow aerobically and tolerate high concentrations of tellurite might be due to the greater redox stability of MSHs as compared to GSHs [49], under oxidative stress conditions generated by the simultaneous presence of oxygen and TeO_3_^2−^. Moreover, catalase, which is a key enzyme that overcomes cellular oxidative stress, is able to reduce tellurite to its elemental form (Te^0^), conferring the resistance to aerobic microorganisms towards this oxyanion [50]. However, the mechanism of tellurite resistance for Gram-positive bacteria belonging to the order of *Actinomycetales* is scarcely studied. Nevertheless, it is noteworthy to mention the study of Terai and coworkers [51], in which a cell free extract of *Mycobacterium avium* was able to reduce tellurite with a non-specific interaction. Furthermore, among tellurite-resistant Gram-positive bacteria, *Bacillus* sp. STG-83 was characterized for its ability to reduce these oxyanions using a cytoplasmic tellurite reductase [52], while the product of the genes *cysK* (cysteine synthase), *cobA* (uroporphyrinogen-III C-methyltransferase), *iscS* (cysteine desulfurase) of *Geobacillus stearothermophilus* V conferred resistance to the *E. coli* K-12 strain towards potassium tellurite [53–55].

The production of intracellular Te-deposits as a consequence of TeO_3_^2−^ reduction was earlier described in Gram-positive bacteria such as *Paenibacillus* TeW and *Salinicoccus* sp. QW6 [22, 23], while Baesman and coworkers reported on the presence of Te-nanostructures in the form of clusters/rosettes accumulated on the outer cell surfaces of *B. beveridgei* and *B. selenitireducens* [16, 17]. In detail, the Te-nanostructures produced by *Bacillus* strains clustered together after their synthesis, forming larger and thicker shard-like structures, which were able to adhere each other and to collapse into bigger rosettes [16, 17]. Conversely, our present TEM images of BCP1 *unconditioned* cells grown in the presence of either 100 or 500 µg/mL of K_2_TeO_3_ revealed the presence of intracellular stable Te-nanorods (TeNRs), similar to those described by Zare and colleagues in *Bacillus* sp. BZ [20]. Moreover, TeNRs isolated from *unconditioned* or *conditioned* BCP1 cells as seen by TEM and SEM analyses, still appeared in the form of individual and not clustered rod-shaped nanostructures (Figs. 4, 6). Isolated TeNRs were embedded into a slightly electron-dense surrounding material, whose organic nature was revealed by signals corresponding to carbon, oxygen, nitrogen and sulfur as detected by EDX spectroscopy. Similar observations were recently obtained by Zonaro and coworkers studying Te-nanoparticles (TeNPs) produced by the Gram-negative *Ochrobactrum* sp. MPV1 strain [26]. The zeta potential measurements highlighted a similar negative potential of either studied TeNRs suspensions or the supernatants recovered from Te-nanostructures (Additional file 1: Figures S3, S4), reinforcing the indication of an organic material associated with the BCP1 TeNRs, possibly involved in stabilizing these nanostructures, since tellurium does not have a net charge in its elemental state (Te^0^). Our conclusion is also in line with the study by Wang et al. [56], who ascribed the strong negative surface potential of chemically synthetized Te-nanowires to carboxylic groups of l-cysteine ligands in solution. Moreover, DLS analyses of all studied TeNRs samples showed size distributions that were virtually indistinguishable for TeNRs extracted from BCP1 *unconditioned* and *conditioned* grown cells. The only factor that appeared to have an effect on the measured sizes was the initial concentration of TeO_3_^2−^ (100 or 500 µg/mL). Additionally, the size distributions of the analyzed supernatants recovered after removing TeNRs showed peaks slightly shifted towards smaller sizes. These results suggest that the size distributions obtained by DLS for all TeNRs suspensions do not depend only on the presence of the nanorods in the samples. Nanostructures are known to have a high surface energy and may be thermodynamically unstable in suspension [57]. The stability of nano-suspensions is increased if there is an electrostatic repulsion between the particles due to the presence of charges on the surface or if the surface is coated with molecules that prevent the particles to come into close contact with each other and collapse into aggregates [58, 59]. The latter form of stabilization, so called steric stabilization, is widely used in chemical synthesis of nanoparticles and nanorods [60]. In the case of TeNRs produced by the BCP1 strain, both electrostatic and steric stabilization seem to play a role. The organic matter surrounding TeNRs is charged as confirmed by zeta potential measurements. It is important to mention that the presence of the organic surrounding material in solution is essential to the stability of TeNRs. Our attempts to remove it from the nanorods suspensions by several rounds of centrifugation resulted in an irreversible aggregation of the TeNRs. This result combined with the DLS and Zeta potential data suggest that (1 the organic surrounding material is not covalently attached to the surface of TeNRs, and (2) it is adsorbed on the surface and also present in solution in equilibrium, playing a crucial role in the colloidal stability of TeNRs. We have not been able to confirm the identy of these organic molecules. However, there is a strong possibility that hydrophobic molecules, either lipids or a secreted biosurfactant may be the major constituents of the mixture. There are at least two arguments in favor of this hypothesis. First, due its amphiphilic properties lipids are known to form nanosized aggregates when suspended in aqueous solution. Such nanostructures were observed by DLS even after the nanorods were removed from solution. Second, chemical synthesis of nanorods typically requires the presence of a surfactant at high concentrations to drive their synthesis to one direction [61]. In this regard, *Rhodococcus* species are known to produce biosurfactant molecules such as trehalose mycolates and glycolipids under physiological and nitrogen limiting growth conditions [62, 63], respectively. Therefore, it is reasonable to suggest that the nanorod formation may be mediated by the biosurfactant co-produced by the BCP1 strain.

Due to the presence of TeNRs embedded in an undefined organic material, the actual length of the nanorods was established using ImageJ software based on TEM images. As a result, an incremented length of TeNRs was observed as function of the tellurite concentration (100 or 500 µg/mL of K_2_TeO_3_), as well as the condition of growth as *unconditioned* or *conditioned* cells. In this regard, the dependence of TeNRs length on the initial concentration of the available precursor (TeO_3_^2−^) was reported for the production of chemically synthesized nanostructures [64], while the variation of nanorods size as function of the growth conditions (*unconditioned* or *conditioned* cells) may be explained by the LaMer mechanism of nanomaterials formation. According to this mechanism, when the reduction of the precursor to its elemental form occurs, a high concentration of monomers in solution is produced, leading to the formation of nucleation seeds that subsequently grow as nanostructures [65]. Most likely, the reduction of the precursor (TeO_3_^2−^) by *unconditioned* BCP1 cells led to the production of a high concentration of monomers (Te^0^) inside the cells, followed by the formation of Te-seeds of nucleation, which finally grew as TeNRs. As a consequence of the *unconditioned* growth, some Te-seeds of nucleation were still present inside the cells re-inoculated to perform the *conditioned* growth, which might be used by *conditioned* cells to produce longer TeNRs.

Several *Rhodococcus* strains were previously described for their ability to generate metal nanostructures i.e. gold (AuNPs) [66], silver (AgNPs) [67], and zinc oxide (ZnONPs) [68] nanoparticles; however, these rhodococci were scarcely investigated as cell factories for the production of metalloid nanostructures. To the best of our knowledge, this is the first report on the synthesis of rod-shaped nanostructures made of elemental tellurium (TeNRs) by a bacterial strain belonging to the *Rhodococcus* genus.

## Conclusions

The capacity of the BCP1 strain belonging to *Rhodococcus* genus to grow aerobically in the presence of high amounts of the toxic oxyanion tellurite and to reduce it into elemental tellurium (Te^0^) was assessed. In particular, *conditioned* BCP1 cells were able to reduce a greater amount of TeO_3_^2−^ oxyanions at a faster rate as compared to *unconditioned* cells. The estimated MIC value (2800 µg/mL or 11.2 mM) of TeO_3_^2−^ for aerobic growth of BCP1 strain underlined its feature to tolerate high concentration of this toxic oxyanion, as compared to other Gram-positive bacteria previously described as tellurite-tolerant and/or resistant microorganisms. Additionally, the BCP1 strain was able to produce intracellular rod-shaped nanostructures, which did not aggregate. These TeNRs were embedded in an organic surrounding material, showing an increasing length as function of tellurite concentration (100 or 500 µg/mL of K_2_TeO_3_) and the growth condition such as *unconditioned* or *conditioned* cells.

Since tellurium is a versatile narrow band-gap p-type semiconductor [69], this element exhibits unique properties such as photoconductivity, high piezoelectricity, thermoelectricity [70], non-linear optical response [71]. In this respect, TeNRs have found applications as optoelectronic, thermoelectric, piezoelectric devices, as well as gas sensors and infrared detectors [72–76]. Moreover, TeNRs have been investigated for their antibacterial, antioxidant and anticancer properties [77]. Although further investigations are required in order to evaluate the potential use of TeNRs synthetized by *Rhodococcus aetherivorans* BCP1, the present study demonstrated that aerobically grown BCP1 strain can be utilized as a cell factory for metalloid nanostructure production.

## Additional file

**Additional file 1.** Additional information.
