# Supplementary material for: Rhodococcus aetherivorans BCP1 as cell factory for the production of intracellular tellurium nanorods under aerobic conditions
Source: Microb Cell Fact. 2016 Dec 15;15:204. doi: 10.1186/s12934-016-0602-8 (PMC5157098; doi:10.1186/s12934-016-0602-8)
Supplement: Supplementary file 1 — Additional file 1. Additional information. [file 12934_2016_602_MOESM1_ESM.docx]

**Supporting information for**

***Rhodococcus* *aetherivorans* BCP1 as Cell Factory for the Production of Intracellular Tellurium Nanorods under Aerobic Conditions**

*Alessandro Presentato*^1,†,*^*, Elena Piacenza*^1,†^*, Max Anikovskiy*^2^*, Martina Cappelletti*^3^*, Davide Zannoni*^3^*, Raymond J. Turner*^1,*^

^†^Contributed equally

^1^*Microbial Biochemistry Laboratory, Department of Biological Sciences, University of Calgary, 2500 University Dr. NW, Calgary, AB T2N 1N4, Canada*

^2^*Department of Chemistry, University of Calgary, 2500 University Dr. NW, Calgary, AB T2N 1N4, Canada*

^3^*Unit of General and Applied Microbiology, Department of Pharmacy and Biotechnology, Via Irnerio 42, Bologna, 40126, Italy*

**Corresponding authors:** Alessandro Presentato, Raymond J. Turner

**Email addresses:**

Alessandro Presentato: [alessandro.presentat@ucalgary.ca](mailto:alessandro.presentat@ucalgary.ca)

Elena Piacenza: [elena.piacenza@ucalgary.ca](mailto:elena.piacenza@ucalgary.ca)

Max Anikovskiy: [m.anikovskiy@ucalgary.ca](mailto:m.anikovskiy@ucalgary.ca)

Martina Cappelletti: martina.cappelletti2@unibo.it

Davide Zannoni: davide.zannoni@unibo.it

Raymond J. Turner: turnerr@ucalgary.ca

**Dynamic Light Scattering (DLS) of tellurium nanorods produced by *Rhodococcus aetherivorans* BCP1 grown as *unconditioned* or *conditioned* cells in the LB medium supplemented with 100 or 500 μg/mL potassium tellurite (Figure S1).**

**Figure S1**: Dynamic Light Scattering (DLS) analysis of TeNRs_100_ (**a and b**), and TeNRs_500_ (**c and d**) extracted from the BCP1 strain grown as *unconditioned* (**a and c**) or *conditioned* (**b and d**) cells in the presence of K_2_TeO_3_.

**Dynamic Light Scattering (DLS) of the supernatants in which tellurium nanorods are suspended. The supernatants are recovered by removing tellurium nanorods through centrifugation, and DLS control experiment of supernatant derived from the culture of BCP1 cells grown for 120 h on rich medium (LB) not exposed to potassium tellurite (Figure S2).**

**Figure S2**: Dynamic Light Scattering (DLS) analysis of supernatants recovered from TeNRs_100_ (**a and b**), and TeNRs_500_ (**c and d**) extracted from the BCP1 strain grown as *unconditioned* (**a and c**) or *conditioned* (**b and d**) cells in the presence of K_2_TeO_3_. DLS plot of the supernatant derived from BCP1 culture grown for 120 h on rich medium (LB) not exposed to K_2_TeO_3_ (**e**).

**Zeta potential measurements of tellurium nanorods recovered from BCP1 biomass grown in the presence of 100 or 500 μg/mL potassium tellurite as *unconditioned* or *conditioned* cells (Figure S3).**

**Figure S3**: Zeta Potential measurements of TeNRs_100_ (a), and TeNRs_500_ (b) generated by *unconditioned* BCP1 cells, and TeNRs_100_ (c), and TeNRs_500_ (d) extracted from *conditioned* BCP1 cells grown in the presence of K_2_TeO_3_.

**Zeta potential analysis of the supernatants recovered from tellurium nanorod samples by centrifugation (Figure S3).**

**Figure S4**: Zeta Potential measurements of the supernatants recovered from TeNRs_100_ (**a**), and TeNRs_500_ (**b**) generated by *unconditioned* BCP1 cells, and those of TeNRs_100_ (**c**), and TeNRs_500_ (**d**) extracted from *conditioned* BCP1 cells grown in the presence of K_2_TeO_3_.
